# Author Correction: Human neocortical expansion involves glutamatergic neuron diversification

**DOI:** 10.1038/s41586-021-04322-4

**Published:** 2022-01-06

**Authors:** Jim Berg, Staci A. Sorensen, Jonathan T. Ting, Jeremy A. Miller, Thomas Chartrand, Anatoly Buchin, Trygve E. Bakken, Agata Budzillo, Nick Dee, Song-Lin Ding, Nathan W. Gouwens, Rebecca D. Hodge, Brian Kalmbach, Changkyu Lee, Brian R. Lee, Lauren Alfiler, Katherine Baker, Eliza Barkan, Allison Beller, Kyla Berry, Darren Bertagnolli, Kris Bickley, Jasmine Bomben, Thomas Braun, Krissy Brouner, Tamara Casper, Peter Chong, Kirsten Crichton, Rachel Dalley, Rebecca de Frates, Tsega Desta, Samuel Dingman Lee, Florence D’Orazi, Nadezhda Dotson, Tom Egdorf, Rachel Enstrom, Colin Farrell, David Feng, Olivia Fong, Szabina Furdan, Anna A. Galakhova, Clare Gamlin, Amanda Gary, Alexandra Glandon, Jeff Goldy, Melissa Gorham, Natalia A. Goriounova, Sergey Gratiy, Lucas Graybuck, Hong Gu, Kristen Hadley, Nathan Hansen, Tim S. Heistek, Alex M. Henry, Djai B. Heyer, DiJon Hill, Chris Hill, Madie Hupp, Tim Jarsky, Sara Kebede, Lisa Keene, Lisa Kim, Mean-Hwan Kim, Matthew Kroll, Caitlin Latimer, Boaz P. Levi, Katherine E. Link, Matthew Mallory, Rusty Mann, Desiree Marshall, Michelle Maxwell, Medea McGraw, Delissa McMillen, Erica Melief, Eline J. Mertens, Leona Mezei, Norbert Mihut, Stephanie Mok, Gabor Molnar, Alice Mukora, Lindsay Ng, Kiet Ngo, Philip R. Nicovich, Julie Nyhus, Gaspar Olah, Aaron Oldre, Victoria Omstead, Attila Ozsvar, Daniel Park, Hanchuan Peng, Trangthanh Pham, Christina A. Pom, Lydia Potekhina, Ramkumar Rajanbabu, Shea Ransford, David Reid, Christine Rimorin, Augustin Ruiz, David Sandman, Josef Sulc, Susan M. Sunkin, Aaron Szafer, Viktor Szemenyei, Elliot R. Thomsen, Michael Tieu, Amy Torkelson, Jessica Trinh, Herman Tung, Wayne Wakeman, Femke Waleboer, Katelyn Ward, René Wilbers, Grace Williams, Zizhen Yao, Jae-Geun Yoon, Costas Anastassiou, Anton Arkhipov, Pal Barzo, Amy Bernard, Charles Cobbs, Philip C. de Witt Hamer, Richard G. Ellenbogen, Luke Esposito, Manuel Ferreira, Ryder P. Gwinn, Michael J. Hawrylycz, Patrick R. Hof, Sander Idema, Allan R. Jones, C. Dirk Keene, Andrew L. Ko, Gabe J. Murphy, Lydia Ng, Jeffrey G. Ojemann, Anoop P. Patel, John W. Phillips, Daniel L. Silbergeld, Kimberly Smith, Bosiljka Tasic, Rafael Yuste, Idan Segev, Christiaan P. J. de Kock, Huibert D. Mansvelder, Gabor Tamas, Hongkui Zeng, Christof Koch, Ed S. Lein

**Affiliations:** 1 Allen Institute for Brain Science, Seattle, WA USA; 2 Department of Physiology and Biophysics, University of Washington, Seattle, WA USA; 3 Department of Pathology, University of Washington, Seattle, WA USA; 4 byte physics, Berlin, Germany; 5 MTA-SZTE Research Group for Cortical Microcircuits, Department of Physiology, Anatomy, and Neuroscience, University of Szeged, Szeged, Hungary; 6 Department of Integrative Neurophysiology, Center for Neurogenomics and Cognitive Research (CNCR), Vrije Universiteit, Amsterdam, The Netherlands; 7 Swedish Neuroscience Institute, Seattle, WA USA; 8 Department of Neurosurgery, University of Szeged, Szeged, Hungary; 9 Cancer Center Amsterdam, Brain Tumor Center, Department of Neurosurgery, Amsterdam UMC, Vrije Universiteit, Amsterdam, The Netherlands; 10 Department of Neurological Surgery, University of Washington, Seattle, WA USA; 11 Nash Family Department of Neuroscience and Friedman Brain Institute, Icahn School of Medicine at Mount Sinai, New York, NY USA; 12 NeuroTechnology Center, Columbia University, New York, NY USA; 13 Edmond and Lily Safra Center for Brain Sciences and Department of Neurobiology, The Hebrew University Jerusalem, Jerusalem, Israel

**Keywords:** Cellular neuroscience, Molecular neuroscience

Correction to: *Nature* 10.1038/s41586-021-03813-8 Published online 6 October 2021

In the version of this Article initially published, the Acknowledgements statement contained an error. Originally appearing with thanks for support given in part as follows, “R01EY023173 from The National Eye Institute, U01MH105982 from the National Institute of Mental Health and Eunice Kennedy Shriver National Institute of Child Health & Human Development, and R011EY023173 from The National Institute of Allergy and Infectious Disease,” the last number (R011EY023173) was mistakenly added and is not in fact a grant or one provided by the NIAID. The mention has been removed.

The changes have been made to the online version of the Article.

