# Supplementary material for: Human neocortical expansion involves glutamatergic neuron diversification
Source: Nature. 2021 Oct 6;598(7879):151–8. doi: 10.1038/s41586-021-03813-8 (PMC8494638; doi:10.1038/s41586-021-03813-8)
Supplement: Supplementary file 1 — This Supplementary Note contains extended description of the electrophysiological and morphological properties of the mouse and human L2-3 glutamatergic t-types. [file 41586_2021_3813_MOESM1_ESM.pdf]

nature portfolio

https://doi.org/10.1038/s41586-021-03813-8

Supplementary information

Human neocortical expansion involves glutamatergic neuron diversification

In the format provided by the authors and unedited

nature portfolio

https://doi.org/10.1038/s41586-021-03813-8

Supplementary information

Human neocortical expansion involves glutamatergic neuron diversification

In the format provided by the authors and unedited

Supplemental note: extended morphoelectric descriptions

The human glutamatergic t-types divide L2-3 into superficial (LTK and GLP2R) and deep (CARM1P1 and COL22A1) regions, with the FREM3 type spanning both. A dividing line at normalized L2-3 depth = 0.58 (consistent with the nadir in the neuron density at depth = 0.575; Fig. 1b) separates these deep and superficial types with 94% accuracy (assessed by a logistic regression classifier). *LTK* neurons were found primarily in L2 and in the border region of L2 and L3. *GLP2R* neurons were found primarily in upper L3, with some neurons found in L2. *FREM3* neurons spanned L2 and L3, continuing into L4, consistent with their heterogeneous gene expression profile. *CARM1P1* and *COL22A1* were found almost entirely in deep L3 and along the L3/L4 border.

LTK neurons were found primarily in L2 and upper L3, and in general exhibited a regular firing pattern with little firing rate adaptation and no sag. Morphologically, *LTK* neurons were relatively short, but extended multiple apical branches into L1. *GLP2R* neurons were found just deeper than *LTK* neurons, primarily in upper L3, and exhibited some electrophysiological features similar to *LTK* neurons, such as lack of adaptation of action potential firing and higher range of input resistance values but differed from *LTK* neurons in that they had pronounced sag. The *GLP2R* neurons tended to have fewer dendritic branches for their longer apical extent, and often had a pronounced apical tuft in L1. The *FREM3* t-type represented 56.7% of supragranular glutamatergic neurons collected in L2 or L3 dissections, with a laminar distribution that spanned the entire distribution of *LTK* and *GLP2R* (and beyond into deep L3 and L4) and had morphoelectric properties that were overlapping but distinct from those t-types. *FREM3* neurons varied from small neurons in upper L2 to very large magnopyramidal neurons in the deeper part of L3 and had a gradient of morpho-electric properties like the graded transcriptional properties described above. Upper L2 *FREM3* neurons had an apical dendrite restricted to L1 and L2 and regular firing while the large, deep L3 *FREM3* neurons had an apical dendrite that spanned supragranular layers and into L1 and exhibited a rapidly adapting action potential firing. The main apical dendrites of *FREM3* and *LTK* neurons branched much closer to the soma than *GLP2R* neurons at comparable depth, resulting in more radial branching across layers. *CARM1P1* neurons were found in deep L3 and exhibited extensive apical oblique and basal dendritic branching near the soma as well as a faster action potential upstroke than other types. *COL22A1* neurons had very sparse basal and apical dendritic branching and exhibited very high input resistance and thus were the most responsive to current injection, displaying a steeper firing frequency to current input gain relative to the other t-types. Interestingly, *COL22A1* neurons showed a smaller amount of sag than *CARM1P1* neurons located at an equivalent distance from pia, indicating that this property is t-type-specific rather than explicitly depth-dependent.

Comparison of human and mouse L2-3 t-types

Each of the three mouse t-types had distributions spanning upper and deep L2/3, although there was a trend for *Adamts2* and *Rrad* to be more superficial. Similarly, each of these t-types contained neurons with wide and tufted branching. To quantify the degree of separation or overlap of t-types in different features between mouse and human, we ran a one-way ANOVA

for the effect of t-type on each calculated electrophysiology and morphological feature. For electrophysiological features (Extended Data Fig. 4), 3/18 showed differences between t-types that explained >10% of feature variance (R^2>0.1) for both human and mouse (FDR<0.05 for mouse features, <10^{-5} for human). However, the mouse types were distinct in input resistance and two related AP shape features (width and downstroke) with a maximum R^2 =0.12, while the human types showed distinct firing properties (f-I slope and rheobase) in addition to input resistance, with a maximum R^2 =0.16. For morphological features (Extended Data Fig. 4), 16/60 features had R^2 >0.15 among the human t-types (FDR<10^{-3}), compared to 12/60 for the mouse t-types (with 10/12 significant at FDR<0.05). This quantitative analysis confirms the qualitative observation that the main supragranular human t-types are more morphoelectrically specialized with respect to one another than their mouse homologues, primarily in terms of morphology, although how much of this is due to areal variation is unknown.
