# Supplementary material for: Human neocortical expansion involves glutamatergic neuron diversification
Source: Nature. 2021 Oct 6;598(7879):151–8. doi: 10.1038/s41586-021-03813-8 (PMC8494638; doi:10.1038/s41586-021-03813-8)
Supplement: Supplementary file 2 — Reporting Summary [file 41586_2021_3813_MOESM2_ESM.pdf]

nature research

Corresponding author(s): Ed Lein

Last updated by author(s): Mar 31, 2021

Reporting Summary

Nature Research wishes to improve the reproducibility of the work that we publish. This form provides structure for consistency and transparency in reporting. For further information on Nature Research policies, see Authors & Referees and the Editorial Policy Checklist.

Statistics

For all statistical analyses, confirm that the following items are present in the figure legend, table legend, main text, or Methods section.

n/a	Confirmed	
☐	☒	The exact sample size (*n*) for each experimental group/condition, given as a discrete number and unit of measurement
☒	☐	A statement on whether measurements were taken from distinct samples or whether the same sample was measured repeatedly
☐	☒	The statistical test(s) used AND whether they are one- or two-sided<br>*Only common tests should be described solely by name; describe more complex techniques in the Methods section.*
☐	☒	A description of all covariates tested
☐	☒	A description of any assumptions or corrections, such as tests of normality and adjustment for multiple comparisons
☐	☒	A full description of the statistical parameters including central tendency (e.g. means) or other basic estimates (e.g. regression coefficient) AND variation (e.g. standard deviation) or associated estimates of uncertainty (e.g. confidence intervals)
☐	☒	For null hypothesis testing, the test statistic (e.g. *F*, *t*, *r*) with confidence intervals, effect sizes, degrees of freedom and *P* value noted<br>*Give P values as exact values whenever suitable.*
☒	☐	For Bayesian analysis, information on the choice of priors and Markov chain Monte Carlo settings
☒	☐	For hierarchical and complex designs, identification of the appropriate level for tests and full reporting of outcomes
☐	☒	Estimates of effect sizes (e.g. Cohen's *d*, Pearson's *r*), indicating how they were calculated

Our web collection on statistics for biologists contains articles on many of the points above.

Software and code

Policy information about availability of computer code

Data collection	Electrophysiology acquisition: Igor Pro 8 (Wavemetrics) with a custom acquisition module (https://github.com/AllenInstitute/MIES).<br>Image acquisition: Zeiss Efficient navigation (ZEN) 2012 SP2 software module (Zeiss).
Data analysis	The Vaa3D version 3.2 morphological reconstruction software, including the Mozak extension, is freely available at www.vaa3d.org and its code is available at github.com/Vaa3D. The Python 3 code for electrophysiological and morphological feature analysis is available as part of open-source repositories (github.com/AllenInstitute/AllenSDK, github.com/alleninstitute/ipfx, github.com/alleninstitute/neuron_morphology).<br>Mapping and other transcriptomic analysis was performed using custom software written in R (3.6.1) which used several packages including cowplot (1.0.0), data.table (1.12.4), dplyr (0.8.3), feather (0.3.5), future (1.14.0), genesorteR (0.4.2), ggbeeswarm (0.6.0), ggplot2 (3.2.1), gplots (3.0.1.1), gridExtra (2.3), Matrix (1.2.17), matrixStats (0.55.0), mfishtools (0.0.1), pheatmap (1.0.12), scrattch.hicat (0.0.23), scrattch.vis (0.0.212), Seurat (3.1.1), umap (0.2.4.1), VENcelltypes (0.1.0), and WGCNA (1.68). Sequence alignment was performed using STAR v2.5.3.<br>Analysis of electrophysiology and morphology data was performed using custom software written in Python (3.7), using several packages including numpy (1.15), pandas (0.2.4), scipy (1.4), scikit-learn (0.22), seaborn (0.9), statsmodels (0.11), and umap-learn (0.3.8).<br>Morphological features were calculated using the version of neuron_morphology package (https://github.com/alleninstitute/neuron_morphology/tree/dev).<br>Soma area and density was calculated in NeuN+ neurons using custom software written in R (3.6.1) which used several packages including dplyr (0.8.3), ggplot2 (3.2.1), msir (1.3.2), and RImageJROI (0.1.1).<br>All custom code will be made available at github.com/alleninstitute/patchseq_human_L23.<br>Analysis of gene-depth correlations was performed using ToppGene (https://toppgene.cchmc.org/enrichment.jsp) and REViGO (http://revigo.irb.hr/), along with custom Python (3.7) software using the goatools package (0.9.9).

For manuscripts utilizing custom algorithms or software that are central to the research but not yet described in published literature, software must be made available to editors/reviewers. We strongly encourage code deposition in a community repository (e.g. GitHub). See the Nature Research guidelines for submitting code & software for further information.

Data

Policy information about availability of data

All manuscripts must include a data availability statement. This statement should provide the following information, where applicable:

- Accession codes, unique identifiers, or web links for publicly available datasets
- A list of figures that have associated raw data
- A description of any restrictions on data availability

Transcriptomic, electrophysiological, and morphological data supporting the findings of this study are available at https://portal.brain-map.org/explore/classes/multimodal-characterization.

Field-specific reporting

Please select the one below that is the best fit for your research. If you are not sure, read the appropriate sections before making your selection.

☒ Life sciences ☐ Behavioural & social sciences ☐ Ecological, evolutionary & environmental sciences

For a reference copy of the document with all sections, see nature.com/documents/nr-reporting-summary-flat.pdf

Life sciences study design

All studies must disclose on these points even when the disclosure is negative.

Sample size	No statistical methods were used to predetermine sample sizes, but the sample sizes here are similar to those reported in previous publications.<br>The size of the final data set was determined based on reaching a number of cells per transcriptomic type on which meaningful statistics could be applied to determine phenotypic differences. For the most rare type we encountered, CARM1P1, this worked out to 5 neurons with intact dendrite morphologies.
Data exclusions	All cells included in this study were used in the transcriptomics analysis and passed the exclusion criteria laid out in the "Dataset curation" Methods section. Addition data exclusions were imposed for electrophysiology and morphology. To be included in electrophysiology analysis, each cell was required to pass a set of pre-established quality control metrics, including a > 1 GΩ seal recorded prior to break-in and the initial access resistance < 20 MΩ and < 15% of the input resistance. For an individual sweep to be included: (1) the bridge balance was < 20 MΩ and < 15% of the Rinput, (2) bias (leak) current 0 +/- 100 pA, (3) root mean square (RMS) noise measurements in a short window (1.5 ms, to gauge high frequency noise) and longer window (500 ms, to measure patch instability) < 0.07 mV and 0.5 mV, respectively and (4) the difference in the voltage at the end of the data sweep (measured over 500 ms of rest) and the voltage measured immediately prior to the stimulus onset < 1 mV. (For some human cells, after inspection of traces by eye, exceptions were made to cell-level QC and the sweep-level constraint on change in voltage relaxed from 1 to 2 mV.)<br>Cells were excluded from morphological reconstruction due to pre-established quality control metrics (poor fill, extensive truncation or breakage of axon or dendrites).
Replication	A total of 385 human Patch-seq cells from 82 individuals were used in the analysis, including cells from multiple types of surgeries and from multiple cortical areas. Data acquired from multiple mice from multiple litters per transgenic line surveyed. Extensive acquisition metadata as well as detailed white papers are reported as part of the Allen Cell Types Database (celltypes.brain-map.org); these additional details are intended to aid other laboratories if they seek to replicate the results presented in this study.<br>Each cell is recorded as an independent experiment and tissue was collected from 90 independent donors. The fact that so cells from so many donots map so reliably (see Extended Data Figure 2) supports the replicability of this study.
Randomization	Randomization is not relevant to this study as there was a single condition for all acquired data.
Blinding	Blinding is not relevant to this study as there was a single condition for all acquired data.

Reporting for specific materials, systems and methods

We require information from authors about some types of materials, experimental systems and methods used in many studies. Here, indicate whether each material, system or method listed is relevant to your study. If you are not sure if a list item applies to your research, read the appropriate section before selecting a response.

Materials & experimental systems

n/a	Involved in the study
☐	☒ Antibodies
☒	☐ Eukaryotic cell lines
☒	☐ Palaeontology
☐	☒ Animals and other organisms
☐	☒ Human research participants
☒	☐ Clinical data

Methods

n/a	Involved in the study
☒	☐ ChIP-seq
☒	☐ Flow cytometry
☒	☐ MRI-based neuroimaging

Antibodies

Antibodies used

Primary mouse anti-Neurofilament H (SMI-32, Biolegend, 801701), Neu-N (Millipore #MAB377, 1:2000); SMI-32 (Biolegend #801704, 1:2000); GFAP (Millipore #MAB360, 1:1500); Parvalbumin (Swant #PV235, 1:2000); Iba-1 (Wako #019-19741, 1:1000); Ki67 (Dako #M724001-2, 1:200), goat anti-mouse IgG (H+L) Alexa Fluor conjugates (594 or 647, ThermoFisher Scientific A-11005 or 21235).

Validation

Anti-Neurofilament H, Neu-N; SMI-32; GFAP; Parvalbumin; and Iba-1 were verified via internal controls in every brain tissue slice - morphologies of expected cells to be stained were verified for each application. Ki67 stains rapidly dividing cells and is not expected to label healthy brain tissue, so a positive control slice was run using tonsil tissue for each application.

Animals and other organisms

Policy information about studies involving animals; ARRIVE guidelines recommended for reporting animal research

Laboratory animals

Mice (male and female) between the ages of P45-P70 were maintained on the C57BL/6J background, and newly received or generated transgenic lines were backcrossed to C57BL/6J.

Wild animals

The study did not involve wild animals.

Field-collected samples

The study did not involve field-collected samples.

Ethics oversight

All procedures were carried out in accordance with Institutional Animal Care and Use Committee at the Allen Institute for Brain

Note that full information on the approval of the study protocol must also be provided in the manuscript.

Human research participants

Policy information about studies involving human research participants

Population characteristics

Humans (male and female) between the ages of 18-85 were that were undergoing surgery for epilepsy (73%), tumor (21%), both epilepsy and tumor (3%), or other reasons (2%) are included in this study. Surgeries were performed on both hemispheres (left=33%; right=67%) and in all four cortical hemispheres, but primarily temporal cortex (85%). Most donors were of unknown (71%) or unspecified (5%) ethnicity, with the following breakdown for individuals of specified ethnicity: 90% Caucasian, 5% non-Hispanic or Latino, 3% Alaskan Native, and 1% African-American. Genetic information is not known.

Recruitment

Surgical specimens were obtained from local hospitals (Harborview Medical Center, Swedish Medical Center and University of Washington Medical Center) in collaboration with local neurosurgeons. Although bias could be present if there are biases in characteristics of individuals undergoing surgery for epilepsy or tumor, or of the subset of individuals undergoing surgery who choose to donor brain tissue, in this study we did not identify any relationship between cell properties and age, gender, neuropathology, or reason for surgery.

Ethics oversight

All patients provided informed consent and experimental procedures were approved by Harborview Medical Center, Swedish Medical Center and University of Washington Medical Center institute review boards before commencing the study.

Note that full information on the approval of the study protocol must also be provided in the manuscript.
